# The 4-Year Experience with Implementation and Routine Use of Pathogen Reduction in a Brazilian Hospital

**DOI:** 10.3390/pathogens10111499

**Published:** 2021-11-18

**Authors:** Roberta Maria Fachini, Rita Fontão-Wendel, Ruth Achkar, Patrícia Scuracchio, Mayra Brito, Marcelo Amaral, Silvano Wendel

**Affiliations:** Hospital Sírio-Libanês Blood Bank, São Paulo 01308-050, Brazil; rita.f.wendel@terra.com.br (R.F.-W.); achkarr@ihsl.com.br (R.A.); scuracchiop@ihsl.com.br (P.S.); britom@ihsl.com.br (M.B.); amaralm@ihsl.com.br (M.A.); snwendel@terra.com.br (S.W.)

**Keywords:** pathogen reduction, blood safety, platelet transfusion, INTERCEPT, plasma

## Abstract

(1) Background: We reviewed the logistics of the implementation of pathogen reduction (PR) using the INTERCEPT Blood System™ for platelets and the experience with routine use and clinical outcomes in the patient population at the Sírio-Libanês Hospital of São Paulo, Brazil. (2) Methods: Platelet concentrate (PC), including pathogen reduced (PR-PC) production, inventory management, discard rates, blood utilization, and clinical outcomes were analyzed over the 40 months before and after PR implementation. Age distribution and wastage rates were compared over the 10 months before and after approval for PR-PC to be stored for up to seven days. (3) Results: A 100% PR-PC inventory was achieved by increasing double apheresis collections and production of double doses using pools of two single apheresis units. Discard rates decreased from 6% to 3% after PR implementation and further decreased to 1.2% after seven-day storage extension for PR-PCs. The blood utilization remained stable, with no increase in component utilization. A significant decrease in adverse transfusion events was observed after the PR implementation. (4) Conclusion: Our experience demonstrates the feasibility for Brazilian blood centers to achieve a 100% PR-PC inventory. All patients at our hospital received PR-PC and showed no increase in blood component utilization and decreased rates of adverse transfusion reactions.

## 1. Introduction

Since the emergence of human immunodeficiency virus (HIV), technological advances have been increasingly introduced to improve blood safety and prevent transfusion-transmitted infections (TTIs) [1]. Several measures have been implemented at various levels, such as careful donor selection, strategies to reduce bacterial contamination, development of sensitive screening assays, and hemovigilance programs [2]. Despite such improvements, the risks associated with bacterial contamination of platelets, viruses, vector-borne pathogens, and emerging infectious diseases remain a problem. 

Transfusion-transmitted bacterial infections (TTBI) and associated transfusion septic reactions are currently the leading cause of transfusion-related morbidity and mortality globally [1,3,4,5,6,7,8,9], although they remain widely under-recognized and under-reported, including in Latin America [1]. Risks associated with viral agents have decreased over the past decades [10]; however, new challenges have arisen with changes in sexual behaviors, social issues, human migrations, and climate change, and vary depending on geographic location. Indeed, disparities in blood safety remain between developed and low/middle-income countries (LMICs). The latter are often burdened by a higher prevalence of infectious diseases, endemic for vector-borne parasitic or viral agents, and are at risk of outbreaks of new emerging infectious diseases (EIDs). Over the past decade, Brazil has experienced recurring or new EID outbreaks of dengue, chikungunya, and Zika viruses [11,12]. Blood donor screening strategies, such as donor health questionnaires and deferrals, as well as blood screening using serological and molecular assays, have contributed to mitigating the risk of TTI [13,14]. While these approaches are used both in endemic and non-endemic countries, there is some evidence that in times of emerging pathogen outbreaks, laboratory tests may not be commercially available, require time to be implemented, and implementation may not be practical in outbreak areas or feasible in resource-constrained environments. The most recent experience with SARS-CoV-2 emergence has shown that, although SARS-CoV-2 may not be transfusion-transmitted, blood continuity can be adversely affected during pandemics [15,16].

Pathogen reduction technology (PRT) is increasingly recognized as a proactive approach to mitigate the risks associated with known and unknown blood-borne pathogens [17]. In Brazil, the only available PRT is the INTERCEPT^TM^ Blood System using amotosalen and UVA light for the ex vivo treatment of platelets and plasma, which was approved by the National Regulatory Agency (ANVISA) in 2015. The same technology is also the only FDA-approved PRT for platelets in the US. It inactivates a wide spectrum of bacteria, viruses, and parasites, including emerging infectious agents, and can be used to safeguard the plasma and platelet supply [18,19,20,21]. This nucleic acid targeting technology causes adduct formation and irreversibly crosslinks nucleic acids, preventing the replication of contaminating leukocytes and pathogens, thus decreasing the risk of TTI [22] and transfusion-associated graft-versus-host disease (TA-GVHD) [23,24,25,26,27]. The main anticipated disadvantage of PR is a cost increase in blood component production; however, the cost of implementation can be offset by gains in efficiency through streamlined production methods as well as the discontinuation of procedures such as irradiation and the replacement of specific screening strategies. The benefits of PRT and its added value to blood safety have been recognized by blood transfusion services and hospitals in several countries, including some LMICs [3,28,29,30].

Our hospital has 479 beds, and treats patients with different diagnoses and complex underlying conditions. The blood bank performs approximately 10,000–11,000 transfusions per year, with platelet transfusion accounting for 30%. Pathogen reduction using the INTERCEPT Blood System™ for Platelets was implemented in March 2017, after which 100% of the platelet components were treated, followed by the implementation of the INTERCEPT Blood System™ for plasma to treat all plasma components since April 2017 (see Supplementary Figure S1). After an initial validation period, data analyses of component production, costs associated with PR, patient clinical outcomes, and transfusion requirements were equally compared over the 40 months before and after PR implementation.

## 2. Results

### 2.1. PR Implementation Feasibility through PC Production Method Adjustments

The PC and plasma parameters before PR implementation were analyzed (Table 1). The target values necessary to perform PR treatment, and the final values obtained after the validation period are presented in Figures S2–S5, and Table S1-supplement. It is possible to observe that we needed to adjust significantly the volumes and, consequently, the platelet concentrations per mL, in order to meet the ideal parameters established by the manufacturer to the treatment and, at the same time, achieve our requirements regarding the platelet therapeutic dose (minimum of 3 × 10^11^ for single apheresis and 0.55 × 10^11^ for random donor PC).

For transfusion issues, it was defined as PC-DOSE, as all components with a total platelet concentration around 3 × 10^11^ platelets. These may have been collected from a single donor by apheresis, or obtained from a pool of 5–6 UNITS of random platelets, where each unit was produced from a whole blood donation.

In the pre-PR period from November 2013 to March 2017, 7777 platelet doses were transfused, including 6695 (86.1%) apheresis PC and 1082 (13.9%) random donor (RDP)-PC. Between 13 March 2017, and 12 July 2020, 6921 platelet doses were transfused, including 6016 (86.9%) apheresis PC and 905 (13.1%) RDP-PC. The proportions of apheresis PC doses and RDP PC doses produced over the pre- and post-PR periods were similar (Table 2).

In the pre-PR period, out of 6695 apheresis units collected, 4050 (60.5%) were single doses and 2645 (39.5%) were double doses. In the post-PR period, double apheresis collections and low-volume apheresis collections increased to allow 80.2% apheresis PC to be treated with INTERCEPT double storage (DS) disposable sets (Table 2). This was possible because, in addition to the 2809 doses already collected as double apheresis, 2013 single apheresis (33.5% of all apheresis) were also treated with the DS set. Of the 896 transfused RDP doses, 485 (54.1%) were treated with DS sets and 411 (45.9%) with LV sets (Table 2).

### 2.2. Meeting Transfusion Demand through Optimized Inventory Management

The demand for routine transfusion of PR-PC was fulfilled during an observational period of 40 months. Only six (6) apheresis PC units and nine (9) RDP PC units, equaling 0.22% of the total (6921 units), had to be transfused without PR treatment under medical emergency release due to emergency situations to treat nine (9) patients with high-risk bleeding (Table 2).

With PR adoption, a better inventory management system was implemented with optimized collections in anticipation of patients’ needs. As shown in Table 3, the discard rate in the pre-PR period was 5.9% for apheresis PCs (400/6818 expired units) and 22.7% for RDP PCs (2013/8858 expired units). In the post-PR period, when the maximum shelf life of the platelets had not yet been extended to seven days, a significant reduction in discard rates was already observed (3.2% for apheresis PR-PCs and 3.0% for RDP PR-PCs, *p* < 0.001, respectively).

In June 2020, ANVISA approved an extension for the storage of PR-PC for up to seven days. Discard rates were compared for PR-PC from June 2019 to March 2020 when the period of storage for PR-PC was up to five days and from June 2020 to March 2021, when the period of storage for PR-PC was up to seven days. Discard rates significantly decreased from 4.7% for apheresis and 2.6% for RDP PR-PC stored for up to five days versus 1.2% for apheresis and 0.4% for RDP PR-PC stored for up to seven days (*p* < 0.001) (Table 4).

**Figure 1:**
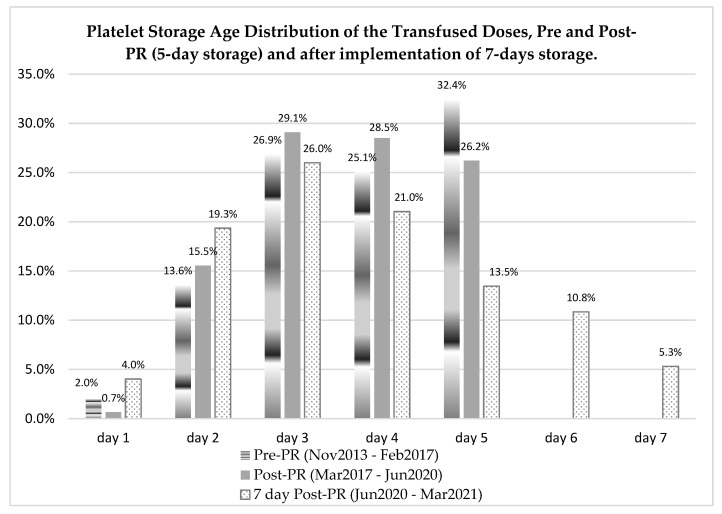
Distribution of platelet transfusions by storage age, in the five (pre- and post-PR) and seven-day storage periods.

The storage ages of the distributed PCs during the different periods are shown in Figure 1. While the average platelet storage duration increased by half a day for the seven-day compared to the five-day period (3.74 ± 1.74 vs. 3.24 ± 1.13 days, respectively, *p* < 0.001), platelet dose availability increased by 16.1%. Interestingly, the mean age of PCs in inventory was reduced from 3.7 ± 1.1 days during the pre-PR implementation period to 3.6 ± 1.1 days during the post-PR implementation period (*p* < 0.001), suggesting an earlier release of PR-PCs. Indeed, the proportion of PCs distributed for ≤3 days was significantly higher during the post-PR period (45.3%) than in the pre-PR period (42.5%, *p* < 0.001). Interestingly, after gaining approval for PR-PC storage extension up to seven days, the mean age of PR-PCs in inventory increased from 3.6 (±1.1) days (SD) back to 3.7 (±1.5) days (SD) (*p* = 0.009), similar to the mean age of PCs in inventory during the pre-PR period 3.7 (±1.1) days (SD) (*p* = 0.621), but the proportion of PR-PCs released ≤3 days was significantly higher (49.4%) than before extension approval (42.5%) (*p* = 0.002). Over the three periods, the proportion of PCs ≥ 4fourdays decreased from 57.5% before PR implementation to 54.7% for PR-PCs up to five days and 50.6% for PR-PCs up to seven days.

It is possible to observe that 16.1% of transfusions had platelets on the sixth and seventh day of collection for the seven-day storage period. Additionally, we can observe that in the period after implementation of seven-days storage (June 2020–March 2021), there was an increase in platelet release with one-day storage. This fact is attributed to the coincidence of this period with the COVID-19 pandemic, which led to some difficulty in the maintenance of regular donors and shorter permanence of blood components in stock for attending the transfusion demand.

### 2.3. Blood Utilization Remained the Same after PR Implementation

Overall, PC utilization did not increase in the post-PR implementation period in comparison to the pre-PR implementation period (5.86 vs. 5.56 units/patients, *p* = 0.5787) nor increased when the PR-PC storage period was increased from up to five days to up to seven days (5.56 vs. 5.19 units per patient, *p* = 0.4737). 

The transfusion demand was studied according to the patient’s profile location: oncology department, intensive care unit (ICU) and emergency room (ER), undergoing surgery, or in the general clinical department. The proportion of transfused doses and the number of patients according to their clinical condition were similar between the pre- and post-PR periods (Table 5). During the pre-PR period, 1031 patients received 7777 PC units, with a mean of 7.5 + 16.5 PC units/patient. Most were men (62.4%), and 60.5-years old on average. Over the post-PR period, patient demographics (60.9% male) and age (mean of 58.1 years) remained stable as did transfusion demand (920 patients received 6921 PC units, with a mean of 7.5 + 20.7 PC units/patient, *p* = 0.36).

The large standard deviations are explained because the Sírio-Libanês Hospital is a reference hospital for high complexity cases, mainly for patients that have already been submitted to successive chemotherapy protocols, and also for patients who are referred for surgical removal of invasive solid tumors, which request high transfusion support during the procedures and in the critical care units.

In highly thrombocytopenic patients, those with pre-transfusion platelet counts lower than 20,000 platelets/μL had a higher number of PC transfusions than those with higher pre-transfusion platelet counts (*p* < 0.0001); in this patient population, the number of PC units/patient did not increase over the post- vs. pre-PR implementation periods (*p* > 0.05) (Table 6). Most patients with pre-transfusion platelet counts ≥ 20,000/µL were transfused due to invasive procedures (oncological, cardiac, or vascular surgeries, liver and cardiac transplants, or interventional radiology procedures with bleeding complications).

In the post-PR implementation period, corrected count increments (CCI) after platelet transfusion were analyzed after the first 60 apheresis PR-PC and 30 RDP PR-PC dose transfusions and platelet recovery for both were considered satisfactory, with no difference observed in mean CCI when compared to the pre-PR implementation period (9038 ± 6599 vs. 10,293 ± 7242, respectively, *p* = 0.41, shown in Figure S6). 

### 2.4. Platelet Transfusion Adverse Events (AEs) Decreased after PR Implementation

There was a significant decrease in the reported AE rate related to platelet transfusions in the post-PR implementation period (1.41% vs. 2.15%, *p* = 0.0008) (Table 7), mainly due to a decrease in mild allergic reactions (1.11% vs. 1.63%, *p* = 0.0065). Although not statistically significant, the frequency of febrile non-hemolytic transfusion reactions (FNHTRs) was lower in the post-PR implementation period (0.26% vs. 0.45%, *p* = 0.058). 

### 2.5. Fresh Frozen Plasma Transfusions

A total of 4119 units of PR-treated plasma (PR-PL) were transfused from April 10, 2017, to August 09, 2020, to 397 patients (10.4 PR-PL units/patient), and in the pre-PR implementation period (10 December 2013, to 09 April 2017), 3301 plasma units were transfused to 516 patients (6.4 units/patient).

This increase in the number of plasma transfusions in the post-PR implementation period was driven by an increase in the number of therapeutic plasma exchange (TPE) procedures (203 sessions of plasmapheresis for TPE treatment in 20 patients vs. 44 sessions in only 5 patients, *p* = 0.0002). 

In the post-PR implementation period, five of the 20 patients who underwent TPE were diagnosed with thrombotic thrombocytopenic purpura (TTP), and all recovered well. Other indications were cardiac and renal transplant rejection (five cases), neurological diseases (three cases), cryoglobulin disease (two cases), bone marrow transplantation (one case) and ABO-incompatible renal transplantation (one case), hemolytic uremic syndrome, Goodpasture syndrome, and thrombocytopenia associated multiple organ failure (TAMOF).

Regardless of the overall increase in patients who underwent plasmapheresis in the post-INTERCEPT period, there was no difference between the two periods in plasma utilization: Pre-PR period: 13.3 units/session vs. 10.5 units/session (*p* = 0.1676) in the post-PR period. There was also no difference between the total of plasma units needed for the treatment of each patient (pre-PR period: mean of 117.4 plasma units per patient vs. 106.8 units in the period post-PR; *p* = 0.8497). 

Excluding the plasma units infused during TPE, 2714 plasma units were transfused to 511 patients (5.31 units per patient) in the pre-PR period, and 1984 plasma units were transfused to 377 patients, (5.26 units per patient) in the post-PR period (*p* = 0.9013), indicating no change in the clinical management of patients requiring plasma transfusion in the periods before and after the introduction of PR treatment.

## 3. Discussion

PR using the amotosalen/UVA PRT was implemented in our service in pursuit of our continued efforts towards increasing blood safety and as a means of preparedness to avoid critical shortages in times of disaster. 

While PR improves blood safety, some reports have described increased costs [31]. We were able to take advantage of these benefits, thus balancing the costs of PR. A direct combination of both was attained at our institution by: (1) replacing selective testing such as cytomegalovirus serology; (2) reducing the need for additional blood screening tests such as bacterial screening of PC [13]; (3) replacing gamma irradiation to prevent TA-GVHD [32,33,34]; and (4) reducing the frequency of platelet transfusion reactions [35,36]. Indeed, the INTERCEPT Blood System, which has a high inactivation efficacy for bacteria, has been recognized by the FDA as an alternative to complex culture-based screening algorithms [5,37,38,39,40,41,42]. As we used to test 100% of all PC by bacterial cultures (BacT/ALERT^TM^), we immediately removed this procedure and its associated costs. In addition, gamma irradiation could be replaced with no detrimental effect to our patients who would be considered at risk for the development of TA-GVHD, and with the added benefit of reducing the risk of TA-GVHD for all patients. Other measures to decrease our costs even further without compromising the quality of the blood components included an increase in double platelet collection and double dose treatment through the implementation of a new product (pool of two single apheresis) and the use of the DS disposable set. A significant decrease in the discarding of platelets was also obtained, highlighting the importance of daily management of platelet inventories for safe stock control and avoiding wastage of such important resources. There was no increase in the demand for platelet or plasma transfusion, demonstrating that PR had no adverse impact on the quality and hemostatic efficacy of the treated components. 

In terms of clinical efficacy, the US SPRINT trial and a European study [28] found significantly lower CCIs and a shorter interval between transfusions with PR-PC by comparing three groups: (1) PR-PC in PAS,(2) untreated PC in PAS, and (3) untreated PC in plasma. Additionally, Janetzko et al. [43] found lower mean one-hour and 24-hour CCIs in PR-PC when compared to untreated apheresis PC, but the differences were not significant. A Cochrane analysis found moderate-quality evidence but no increase in clinically significant bleeding complications (WHO Grade > 2A) [44]. The same analysis found high-quality evidence that patients who received PR-PC required more platelet transfusions, probably due to a shorter time between transfusions and a significantly lower 24-h count increment. However, evidence suggests that PR-PC does not increase the risk of bleeding, death, or serious AEs. Furthermore, a recent study showed no increase in alloimmunization and refractoriness in INTERCEPT-treated platelets [40].

The findings of this study demonstrate that the transfusion efficacy of both PR-PC and PC (all resuspended in 100% plasma) is the same for treating patients with similar underlying conditions and diagnosis [oncology department, intensive care units (ICU) and emergency room (ER), under surgical circumstances or general clinical department)]. The proportion of transfused doses and number of patients by group of pathology or clinical condition were similar in the pre- and post-PR implementation periods. Additionally, the blood utilization per patient group was similar (*p* > 0.05).

A limitation of our study is that we didn’t study physiological impacts of the PR-PC in terms of hemostasis and inflammation; furthermore it was not our scope to detect impacts on leukocytes, mitochondrial DNA or miRNA. We have only analyzed the platelet utilization based on the number of doses transfused per patients, in both groups, pre- and post-PR. Additionally, we reaffirm that the technology was implemented to mitigate the residual risk of known and/or emerging pathogens.

Another benefit gained with PR-PC was the option to increase the storage age from five to seven days, allowing better inventory management and a lower discard rate. Other studies have demonstrated that six- to seven-day-stored platelets are non-inferior to two- to five-day-stored platelets (CCIs comparison) [45]. The authors did not detect differences between bleeding events and intervals between transfusions, suggesting that these differences may not be clinically apparent or significant [46]. Similarly, Aubron et al., in a recent systematic review [47], demonstrated that although fresher units (<2–3 days of storage) consistently resulted in higher CCIs than older units in hematology-oncology patients, no impact on bleeding events or other clinically relevant associations were identified. Infanti et al. [29] described a retrospective two-cohort study on RBC and PC utilization at a university hospital in Switzerland for two consecutive five-year periods with either 0- to five-day-old conventional PC or 0- to seven-day-old PR-PC with PC issued for transfusion on a “first in, first out” basis. Similar to our findings, the authors reported that the implementation of PR-PC led to a reduction in platelet wastage (from 8.7% to 1.5%). Transfusion of PR-PC more than five days old compared with five days old or less did not increase platelet and RBC use on the same or next day and did not increase transfusion reactions. The authors also reported that CCIs for PR-PC stored for ≤five days were 22.6% lower than for conventional PC and that CCIs declined with increasing storage duration for both PC types, but there was no evidence of increased platelet and RBC utilization with PR-PC > 5 days old compared with ≤5 days old. Furthermore, the mean number of PCs used per patient and duration of PC support were not different for hematology/oncology, allogeneic and autologous hematopoietic stem cell transplant, and general medical/surgical patients, who used the majority of PR-PC. In our study, we evaluated the demand for platelet transfusion after seven-day storage versus five-day storage and no increase in utilization was observed, demonstrating similar clinical effectiveness.

We observed a lower occurrence of allergic reactions with the transfusion of PR-PC. A similar observation was demonstrated by Mertes et al. [36] in a large-scale retrospective study involving almost two million PC transfusions in France, confirming that the type of PC (apheresis vs. pooled buffy coat) and their further processing (PC in native plasma, PC in PAS, PR-PC, gamma irradiation) affects the risk of hypersensitivity transfusion reactions (HTR). A decreased incidence of HTR with PAS as a storage solution has been reported. Interestingly, the lowest incidence of HTR was reported with PR-PC in PAS, while an elevated chance for HTR was found with irradiated apheresis PC.

Based on our analysis, we agree with the review conclusions presented by McCullough et al. on outcomes from randomized clinical trials using pathogen reduced platelets [48]. There are a number of considerations for future studies on PR blood components with regard to the hypothesis that PR platelets would not be clinically inferior to untreated platelets. The authors propose an analytical approach as a complementary methodology to the well established procedures used for systematic reviews of the clinical trials results. For this, it will be important to define a bleeding outcome that is clinically relevant (from patient and physician perspectives), standardize bleeding evaluations and hemovigilance programs, perform random clinical trials, specifically in pediatric and surgical patients, as well as to compare PR-RDP and PR-apheresis platelets.

Finally, it is important to highlight relevant benefits related to better inventory management, with greater flexibility of scheduling donors, optimizing the donation agenda based on donors ABO typing and platelet peripheral counts, in order to enable the priority use of a DS device, and less wastage of PC due to expiration, even in the period when the acceptable expiration for the PR-PC was five days. In the last months, extending the storage of PR-PC for up to seven days resulted in even further decreased wastage rates in our service.

## 4. Materials and Methods

A retrospective two-cohort study of PC production, inventory management, discard rates by expiration date, and clinical usage were analyzed in two consecutive 40-month periods: pre-PR (November 2013 through February 2017) versus post-PR implementation (March 2017 through July 2020). After PR implementation for 100% of the PC inventory, gamma irradiation and bacterial screening by BacT/ALERT™ testing, which was performed for 100% of PCs in the pre-PR period, were discontinued. Other strategies have been implemented to reduce the cost of PR-PC production.

The primary goal was a decrease in the discard rate by expiration date, and to accomplish this objective we applied the following strategies: (a) strict control of the collection agenda for apheresis collections based on anticipated needs; (b) definition of the number of random donor platelets (RDP) for PR treatment to avoid unnecessary loss; and (c) PR-PC issued for transfusion on a “first in, first out” basis. All these measures were based on a close assessment of patients transfused in the previous five-day period in an attempt to project the platelet transfusion demand expected over the following days. 

As a secondary interest, the clinical impact of PC transfusion and adverse events in various patient categories were compared between the pre- and post-PR implementation periods.

In addition, as the Brazilian National Regulatory Agency allowed for an extension of the PR-PC storage period from five days to seven days in June 2020, a supplementary analysis was performed over two independent 10-month periods comparing discard rates and clinical outcomes of PR-PC stored for up to five days (June 2019–March 2020) versus PR-PC stored for up to seven days (June 2020–March 2021). 

### 4.1. Apheresis Preparation

Single and double apheresis PC doses in 100% plasma were collected using a Trima^®^ apheresis (Terumo^®^) device, with the following parameters: target platelet dose of 3.3 × 10^11^ and volume of 275 mL. For single apheresis collections treated as pools of two, we targeted a platelet dose of 3.3 × 10^11^ and a volume of 205 mL, whereas for double dose collections, a platelet dose of 6.6 × 10^11^ and a volume of 410 mL were targeted. 

### 4.2. Single Dose Pooling to Double Dose (New Product)

To decrease the costs of PR-PC production, double apheresis collections were increased, and dual storage (DS) containers were used to produce double platelet doses. Parameters for single apheresis collections were set to enable the pooling of two ABO identical “single-apheresis” for treatment with DS containers. To achieve this, it was necessary to increase the number of double apheresis collections through proper donor recruitment and management. Two single doses of ABO-identical apheresis were pooled using a sterile connection device immediately before PR treatment. The final volume and number of platelets were determined according to the manufacturer’s requirements (Table 1). 

### 4.3. Preparation of Single and Double Dose RDP Concentrates for PR Treatment

Whole blood collections (WBCs) (450 mL) were processed into RDP using the platelet-rich plasma method. After preparation and resting overnight in a platelet incubator, pools of five to six or 10-11 RDP were transferred via a sterile connection into a 600 mL standard transfer. Aggregate-free pools were leukoreduced using a Fresenius^®^ BioP platelet filter (Fresenius Kabi AG, 61346 Bad Homburg, Germany).

### 4.4. Preparation of Fresh Frozen Plasma

The WBCs were held on cooling plates (1.4 butanediol) for up to 2 h to 6 h (maximum) after donation. Then, WBCs were separated into red cells, platelets, and plasma using a Compomat^®^ G5 separation device. Plasma units were immediately frozen in a local freezer at −30 °C. After freezing for a minimum of 12-h, three ABO identical plasma units were thawed (Helmer Scientific^®^) and pooled into a 600 mL transfer container using a sterile connection device (Fresenius Kabi^®^, São Paulo, Brazil). The volume was adjusted within a range of 490–650 mL.

### 4.5. Pathogen Reduction Treatment

#### 4.5.1. For Platelets

Following the manufacturer’s instructions (Cerus Corporation^®^–Operator Manual–v.5, Concord, NH, USA), each platelet concentrate (apheresis or RDP) was sterile-connected to the INTERCEPT set (LV = large volume set for single platelet dose treatment or DS=dual storage container for double platelet dose treatment). The platelet concentrates (PCs) flowed by gravity through the amotosalen (S-59) container into the illumination bag. INT100 delivered the UVA treatment to each unit. Following illumination, the treated PC was passed through gravity into a compound adsorption device (CAD). Incubation in the CAD takes 16–24 h under continuous agitation to remove residual amotosalen and photo products. After CAD treatment, the product is passed through gravity into the storage container(s). Double-dose PCs were split equally into two integrated storage containers. The INTERCEPT PCs were stored for a maximum of five to seven days in a platelet incubator (Melco^®^ Engineering) at 22 ± 2 °C under continuous agitation.

#### 4.5.2. For Plasma

Following the manufacturer’s instructions (Cerus Corporation^®^–Operator Manual–v.5, Concord, CA, USA), each plasma pool was sterile-connected to the INTERCEPT Processing Set for Plasma. The procedure was the same as that described above for platelets, although there was no CAD incubation time. The unit was immediately re-frozen at −30 °C with an expiration date of two years after the collection date.

### 4.6. QC Tests Performed for Blood Components

Platelet counting was performed after a 1:3 dilution in PBS using an automatic hematological instrument (Sysmex^®^, KX-21N, Kobe, Japan). The pH at 22 °C was measured using a pH meter (Thermo Scientific Orion^®^ model 410-A). A platelet corrected count increment (CCI) at 1h to 8h was performed after each transfusion for 90 platelet transfusions (60 plateletpheresis and 30 RDP).

### 4.7. Statistical Analysis

Descriptive analyses were conducted for demographic and clinical variables. Continuous variables were summarized by means and standard deviations, and categorical variables by frequencies and proportions based on non-missing data. Frequencies and proportion parameters before and after PR implementation were compared using Fisher’s exact test; Student’s *t*-test was also performed for comparison of means. Statistical significance was set at *p* < 0.05. All statistical calculations were performed using the Stata-15 statistical package (College Station, TX, USA), except for the analysis related to PC age distribution in inventory across three periods (pre-PR implementation, post-PR implementation for PR-PCs stored for up to five days and for PR-PCs stored for up to seven days), which was performed with SAS 9.4, with *p*-values based on the chi-square test comparing each of the three pairs).

### 4.8. Ethical Declaration

According to Brazilian Resolution 510/2016, surveys with databases whose information is aggregated without the possibility of individual identification do not need the Clinical Research Ethics Committee’s evaluation and approval.

## 5. Conclusions

In conclusion, to fully evaluate the societal perspective of implementing PR, the increase in costs must be weighed against the expected benefits [48]. In addition, the potential cost savings realized with PR implementation can be substantial and need to be evaluated for each institution [49]. We believe that the cost of PR implementation may be offset by production method adjustments, inventory management optimization, and certain tests and procedure replacements.

## Figures and Tables

**Table 1:** Quality control data before introduction of INTERCEPT treatment (pre-PR), target values necessary (grey cells) for INTERCEPT treatment (according to manufacturer’s instructions), and data from post validation period after PR implementation (post-PR).

Product	Study Phase	Volume (mL)	Platelet Concentration (×10^9^/L)	Platelet Dose (×10^11^)	pH (22 °C) *
Single dose platelet apheresis for individual treatment	Pre-PR	225 ± 8	1700 ± 222	3.8 ± 0.5	7.25 ± 0.13
	Target values	255–420	-	2.5–5.0	-
	Post-PR	264 ± 6	1599 ± 122	4.2 ± 0.3	7.18 ± 0.20
	*p*	<0.01	0.03	<0.01	0.11
Single dose platelet apheresis for pool treatment	Pre-PR	NA	NA	NA	NA
	Target values	200–210	-	2.5–4.0	-
	Post-PR	206 ± 5	1868 ± 97	3.5 ± 0.3	7.30 ± 0.20
Double dose platelet apheresis	Pre-PR	447 ± 9	1744 ± 178	7.8 ± 0.8	7.26 ± 0.12
	Target values	375–420	-	2.5–8.0	-
	Post-PR	411 ± 5	1849 ± 97	7.6 ± 0.4	7.27 ± 0.30
	*p*	<0.01	<0.01	0.23	0.86
Random donor platelets (RDP)	Pre-PR	61 ± 2	1308 ± 185	0.8 ± 0.1	7.40 ± 0.10
	Target values	40–45	-	0.7–0.9	-
	Post-PR	43 ± 3	1764 ± 470	0.8 ± 0.2	7.30 ± 0.30
	*p*	<0.01	<0.01	0.24	0.09
Plasma	Pre-PR	240 ± 30	-	-	-
	Target values	150–300 (per unit)	-	-	-
	Post-PR	196 ± 3	-	-	-
	*p*	<0.01			

* pH measured on day 0 or 1. NA: not available. *p* < 0.05 was considered statistically significant.

**Table 2:** Distribution of platelet transfusions (apheresis and RDP) during pre-PR and post-PR implementation.

	Pre-PR (November 13–March 17)		Post-PR (March 17–July 20)		INTERCEPT Device	
					LV	DS
Total platelet transfusions (doses)	**7777**	**100%**	6921	100%	1599 (23.1%)	5307 (76.9%)
Apheresis doses	6695	86.1%	6016 *	86.9%	1188 (19.8%)	4822 (80.2%)
*-Single apheresis* *(single dose)*	4050	60.5%	1188	19.8%	1188 (37.1%)	NA
*-Single apheresis* *(for pool of 2)*	NA	NA	2013	33.5%	NA	2013 (62.9%)
*-Double apheresis*	2645	39.5%	2809 ^&^	46.7%	NA	2809 (100%)
RDP doses	1082	13.9%	905 ^#^	13.1%	411 (45.9%)	485 (54.1%)

Legends: LV, large-volume device; DS, dual storage device; RDP, random donor platelets; NA, not applicable. The proportion of transfused apheresis doses was similar in both periods (*p* = 0.1389). ^&^ The proportion of platelet double doses collected by apheresis was significantly higher in the post-PR period than in the pre-PR period (*p* < 0.001). A new product (two low-volume apheresis pools) was created in the post-PR period, allowing an increase in the use of DS devices for apheresis treatment. For RDP, the devices were used equally. There were, respectively, six (6) apheresis (*) and nine (9) RDP doses (^#^) (0.22% of total doses transfused) that were not treated by PR, due to low blood supply and lack of time for PR treatment for nine (9) patients with extremely urgent bleeding.

**Table 3:** Frequency of apheresis (doses) and RDP (individual units) discarded by five-day storage period before (pre-PR) and after PR implementation (post-PR).

	Pre-PR		Post-PR		
	November 2013–February 2017		March 2017–July 2020		*p*
Component	Production	Discarded (%)	Production	Discarded (%)	
Apheresis	6818	400 (5.9)	6161	196 (3.2)	<0.001
RDP	8858	2013 (22.7)	5485	166 (3.0)	<0.001

A significant reduction was observed in the number of discarded platelets in the post-PR period (*p* < 0.001) for apheresis and RDP.

**Table 4:** PR-treated apheresis and RDP discarded after five vs. seven-day storage period in a subgroup analyzed (10 months).

	Stored for Up to Five Days (June 19–March 20)		Stored for Up to Seven Days (June 20–March 21)		*p*
Component	PR-Treated	Discarded (%)	PR-Treated	Discarded (%)	
Apheresis	1270	59 (4.7)	1526	18 (1.2)	<0.001
RDP	858	22 (2.6)	1375	6 (0.4)	<0.001

Even a lower significant reduction (*p* < 0.001) was observed in the discard of PR-treated platelets stored for up to seven-days for apheresis and RDP (numbers in bold).

**Table 5:** Total number of doses of platelet transfusions analyzed by the clinical department, where patients were located and compared with the pre- and post-PR for a 40-month period.

Clinical Department	Pre-PR November 2013–February 2017			Post-PR March 2017–July 2020			P (Doses)
	Doses (%)	Patients (%)	Dose per Patient (Mean ± sd)	Doses (%)	Patients (%)	Dose per Patient (Mean ± sd)	
**Oncology**	4109 (52.8)	366 (35.5)	13.2 ± 24.8	3,369 (48.7)	282 (30.6)	14.1 ± 32.8	0.45
**Critical**	2774 (35.7)	313 (30.4)	5.0 ± 7.6	2,750 (39.7)	330 (35.9)	4.6 ± 7.6	0.46
**Surgical**	416 (5.4)	226 (21.9)	3.0 ± 6.0	394 (5.7)	206 (22.4)	4.0 ± 14.0	0.75
**Clinical**	478 (6.1)	126 (12.2)	5.4 ± 9.9	408 (5.9)	102 (11.1)	6.2 ± 12.0	0.72
**Total**	7777 (100%)	1031 (100%)	7.5 ± 16.5	6,921 (100%)	920 (100%)	7.5 ± 20.7	0.90

Legend: platelet dose = 3.0 × 10^11^ platelets; D/P: platelet dose/patient (mean ± sd). No clinical difference (*p* > 0.05) was observed between pre-PR and post-PR platelet transfusions in any of the clinical departments analyzed.

**Table 6:** Comparison between pre-PR and post-PR in two groups of PC transfusions separated by pre-transfusion platelet count (< or ≥20,000/µL).

	Pre-PR November 2013–February 2017			Post-PR March 2017–July 2020		
Platelet count pre-transfusion (platelets/µL)	Doses (n)	Patients (n)	Doses/Patient mean + sd (Min-Max)	Doses (n)	Patients (n)	Doses/Patient mean+ sd (Min-Max)
<20,000	3942	513	7.68 + 13.0 (1–178)	3172	412	7.70 + 13.4 (1–158)
>20,000	2778	613	4.53 + 9.2 (1–154)	2753	552	4.99 + 16.9 (1–305)
Total analyzed *	6720			5925		

Legend: * It wasn’t possible to analyze the total of 7777 and 6921 transfusions of the pre- and post-PR periods, respectively, because neither all request in emergency situations, present the platelet count pre-transfusion. The number of doses per patient within each group (< or ≥20,000/µL) was similar in the pre- and post-PR implementation for both groups (*p* > 0.05).

**Table 7:** Adverse events rates by period, pre- and post-PR.

	Pre-PR November 2013–February 2017		Post-PR March 2017–July 2020		*p*
Mild allergic	127	1.63%	76	1.11%	0.0065
FNHTR (*)	35	0.45%	18	0.26%	NS
HTR	2	0.03%	0		NS
Fluid Overload	2	0.03%	0		NS
TRALI	0		1	0.01%	NS
Non-concluded	1	0.01%	2	0.03%	NS
Total	167	2.15%	97	1.41%	0.0008

All components were leukoreduced (*); NS, not significant; FNHTR, febrile non-hemolytic transfusion reaction; HTR, hemolytic transfusion reaction; TRALI, transfusion-related acute lung injury. A significant reduction in the mild allergic rate during the PR period was observed.

## Supplementary Materials

The following are available online at https://www.mdpi.com/article/10.3390/pathogens10111499/s1. Figure S1. Timeline for the stages of validation and implementation of pathogen reduction for blood components in our service, and when the Brazilian Healthy Nacional Agency allowed the extended platelet storage age for up to 7 days. Figure S2. Volume and number of platelets of single apheresis for the period before (2016–A, left) or after PR (B, right). Dark blue and red triangles denote results outside or appropriate for pathogen reduction treatment (PR), respectively, showing that only 0.2% of collected single apheresis would be considered adequate for PR treatment, according to manufacturer’s instructions in the period before treatment, rising to 94.6% after adequate processing changes, where only 2.7% components required further manipulation to be adequate for PR (addition or reduction of volume). Figure S3. Volume and number of platelets of single apheresis for the period before (2016–A, left), final results after PR (B, right). Dark blue and red triangles denote results outside or appropriate for pathogen reduction treatment (PR), respectively, showing that only 1.1% would be adequate for treatment before changes in the processing routine; rising to 79% adequate data after implementation; (21% required addition of plasma to complete adequate volume or reduction of volume due to high number of platelets and/or plasma collected). The final results demonstrate that only 3.8% were not ideal but still acceptable as PR. Figure S4. Volume and number of platelets of pool of 2 plateletpheresis (new product): Dark blue and red triangles denote results outside or appropriate for pathogen reduction treatment (PR), respectively. Only 8.3% of the pools required manipulation before PR. Figure S5. Volume and number of platelets of random donor platelets (RDP) before (A, left) or after changes in the processing routine (B, right). All RDPs were initially inadequate for treatment, rising to 99% thereafter, where 78% of them were effectively treated. Figure S6. Recovery of CCI after platelet transfusion (apheresis = 60 transfusions; pool of random platelets = 30). No difference was observed between them. Both values were considered adequate. Table S1. Changes performed in each department of the Blood Bank, necessary to implementation of INTERCEPT treatment for platelet apheresis, random donor platelets and plasma.
