# Supplementary material for: The 4-Year Experience with Implementation and Routine Use of Pathogen Reduction in a Brazilian Hospital
Source: Pathogens. 2021 Nov 18;10(11):1499. doi: 10.3390/pathogens10111499 (PMC8621808; doi:10.3390/pathogens10111499)
Supplement: Supplementary file 1 [file pathogens-10-01499-s001.zip › pathogens-1456765-supplementary.pdf]

pathogens

Supplementary Materials:

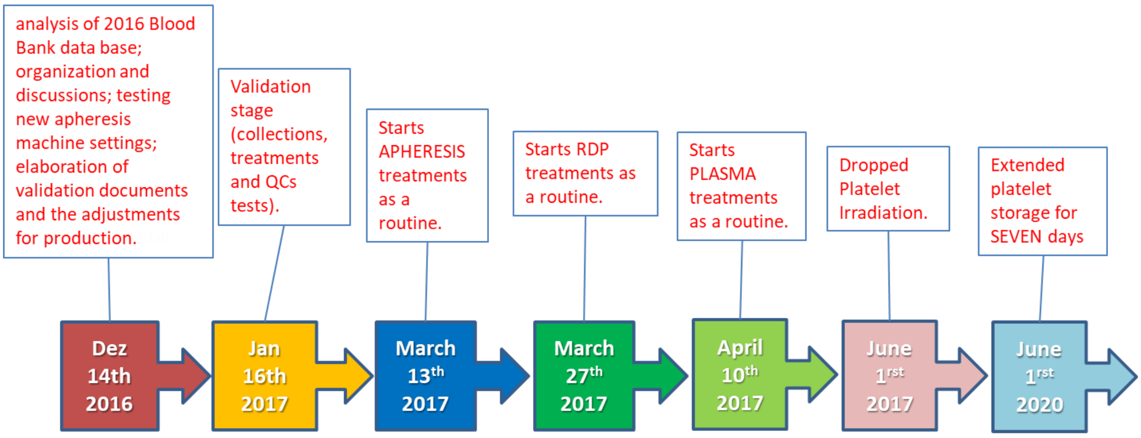

Figure S1. Timeline for the stages of validation and implementation of pathogen reduction for blood components in our service, and when the Brazilian Healthy Nacional Agency allowed the extended platelet storage age for up to 7 days.

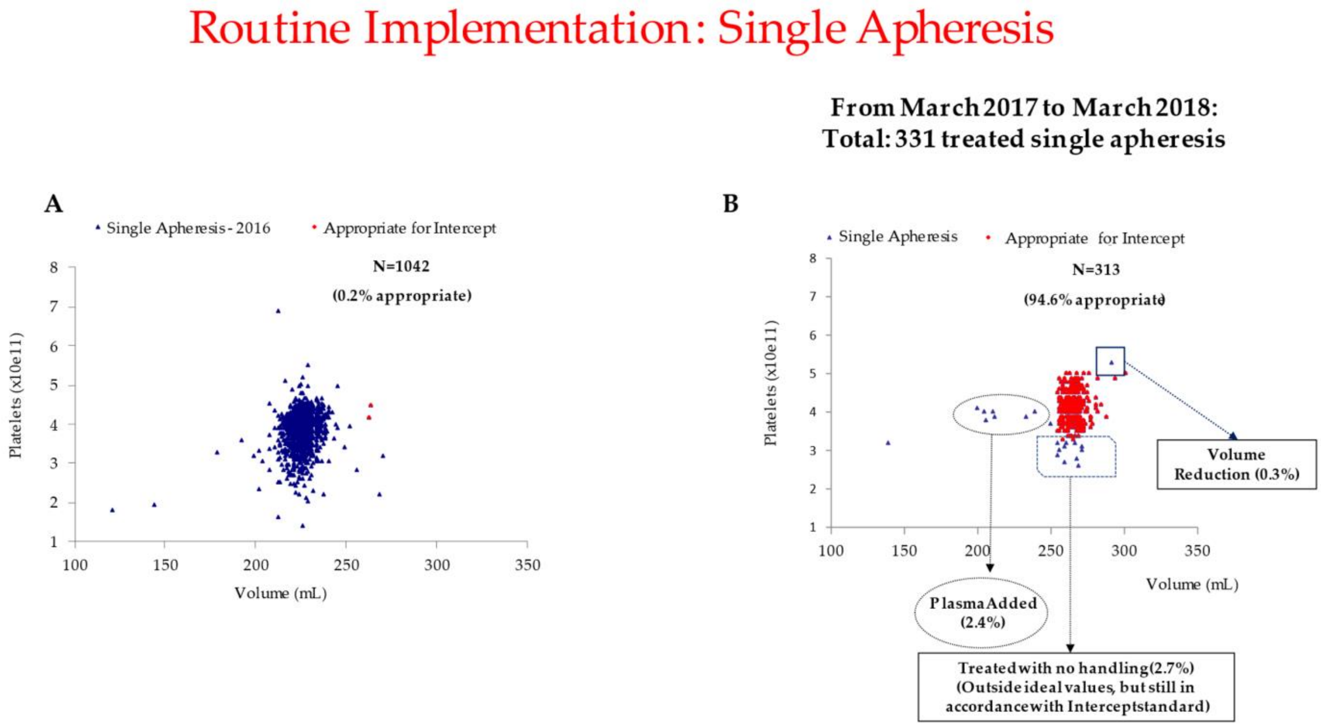

Figure S2. Volume and number of platelets of single apheresis for the period before (2016 – A, left) or after PR (B, right). Dark blue and red triangles denote results outside or appropriate for pathogen reduction treatment (PR), respectively, showing that only 0.2% of collected single apheresis would be considered adequate for PR treatment, according to manufacturer´s instructions in the period before treatment, rising to 94.6% after adequate processing changes, where only 2.7% components required further manipulation to be adequate for PR (addition or reduction of volume).

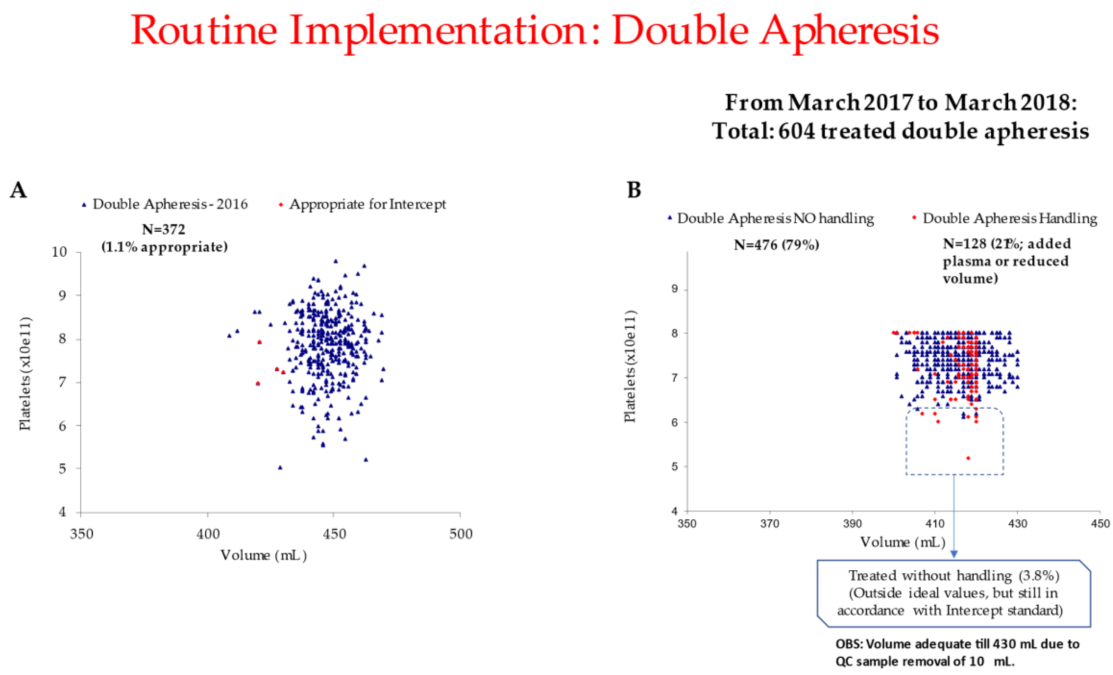

Figure S3. Volume and number of platelets of double apheresis for the period before (2016 – A, left), final results after PR (B, right). Dark blue and red triangles denote results outside or appropriate for pathogen reduction treatment (PR), respectively, showing that only 1.1% would be adequate for treatment before changes in the processing routine; rising to 79% adequate data after implementation; (21% required addition of plasma to complete adequate volume or reduction of volume due to high number of platelets and/or plasma collected). The final results demonstrate that only 3.8% were not ideal but still acceptable as PR. All double apheresis were prepared to remain with the maximum final concentration of 8 × 10^{11}, considering this as the ideal value for the INTERCEPT treatment.

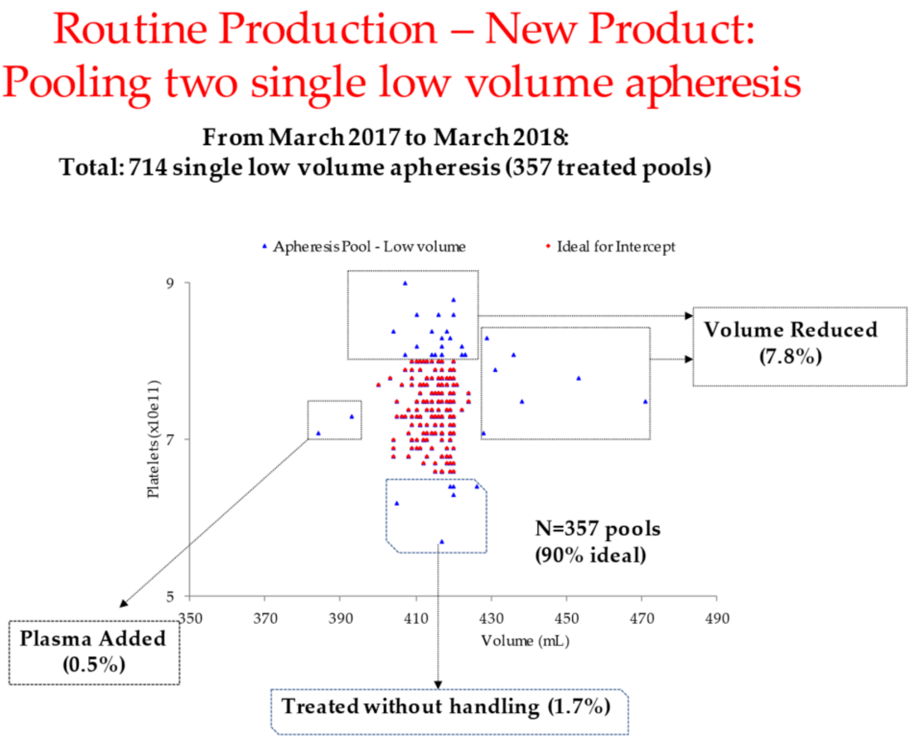

Figure S4. Volume and number of platelets of pool of 2 plateletpheresis (new product): Dark blue and red triangles denote results outside or appropriate for pathogen reduction treatment (PR), respectively. Only 8.3% of the pools required manipulation before PR.

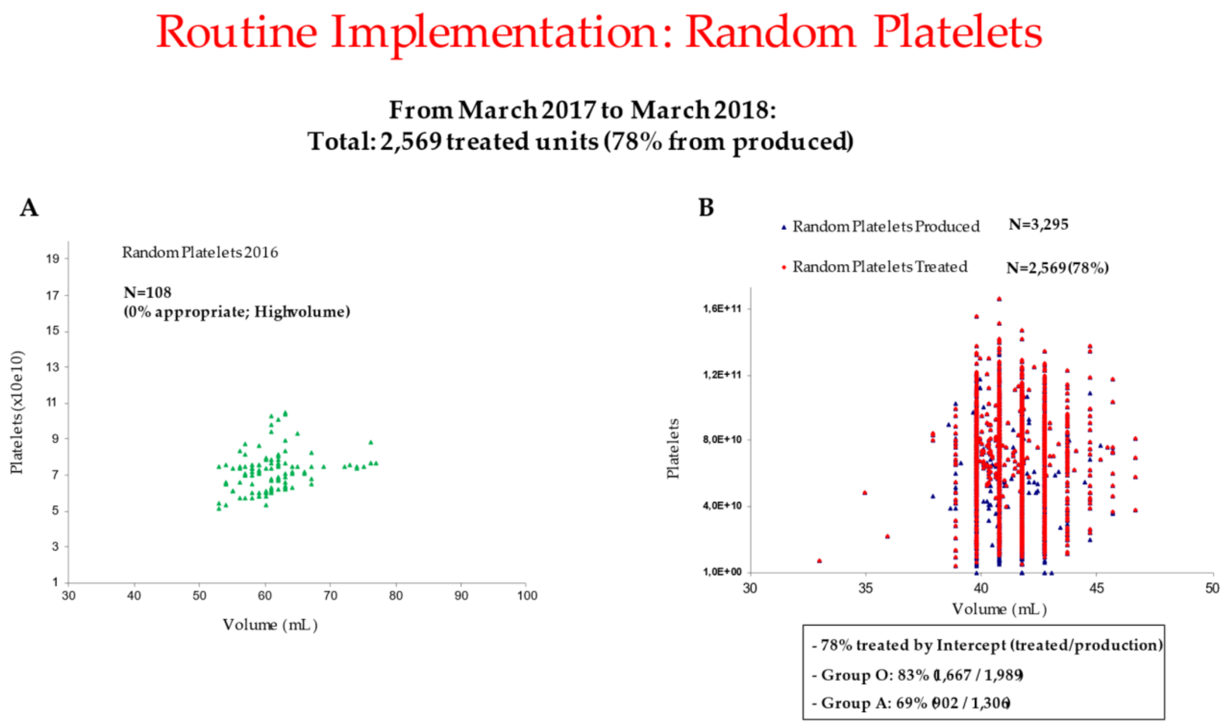

Figure S5. Volume and number of platelets of random donor platelets (RDP) before (A, left) or after changes in the processing routine (B, right). All RDPs were initially inadequate for treatment, rising to 99% thereafter, where 78% of them were effectively treated.

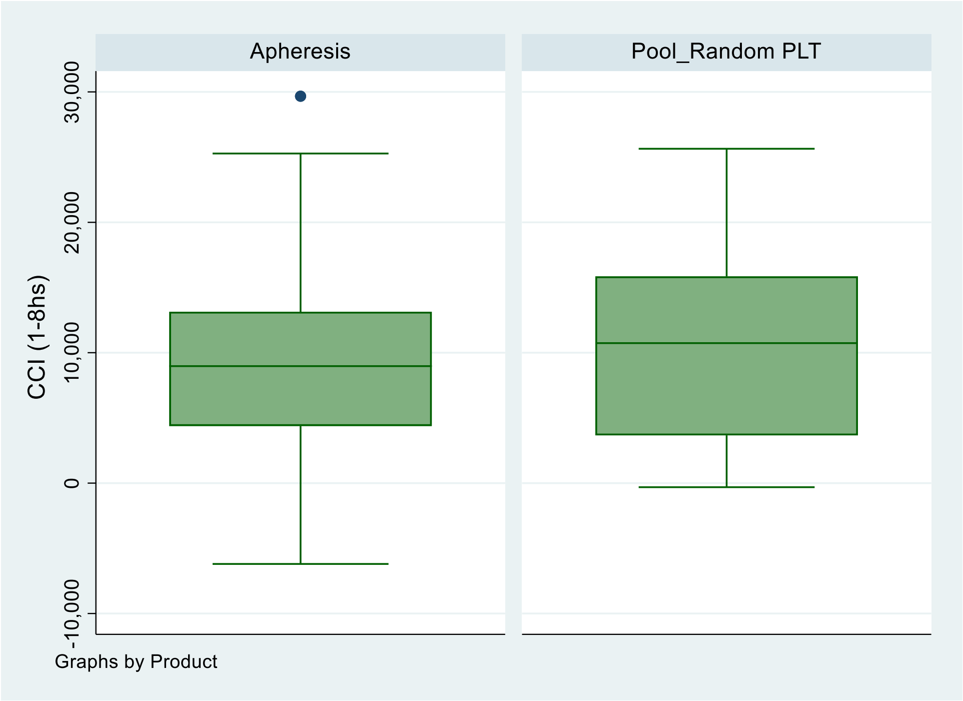

Figure S6. Recovery of CCI after platelet transfusion (apheresis = 60 transfusions; pool of random platelets = 30). No difference was observed between them. Both values were considered adequate.

Table S1. Changes performed in each department of the Blood Bank, necessary to implementation of INTERCEPT treatment for platelet apheresis, random donor platelets and plasma.

<table>
<tr><th>Area involved</th><th>Platelet Apheresis</th><th>Random Donor Platelets (RDP)</th><th>Plasma</th></tr>
<tr><td>Recruitment</td><td>Apheresis only in the morning (weekdays and Saturday).
Collection of group A and O (B and AB only by medical requirement).</td><td>RDP can be collected in the whole day in the weekdays, and only in the Saturday morning.
Collection of group A and O (B and AB only by medical requirement).</td><td>No changes.</td></tr>
<tr><td>Collection</td><td>Modifying and validating apheresis collection parameters: adjusting new volume and number of collected platelets per procedure (together with Terumo®).</td><td>No changes.</td><td>No changes.</td></tr>
<tr><td>Processing lab</td><td colspan="2">Changing in the working time – focus in the evening period, up to 23:00 hours.
Inclusion of a new routine on Sunday morning (due to CAD removal).
Remodelling the processing lab area (minor expansion) due to new machines.
Adjustment on volumes of RDP (Compomat adjustments – together with Fresenius Kabi®).</td><td>Defining time and temperatures for thawing plasmas.
Definition of minimum safety stock for treatment.
Increased expiration data for 2 years.</td></tr>
<tr><td>Quality control Lab</td><td colspan="2">Platelet counts on every collected component (apheresis and random donor platelets).</td><td>No changes.</td></tr>
<tr><td>Information Technology</td><td colspan="3">Redefinition of identification labels.
Readjustments for both blood bank (Hematos) and hospital software system (TASY), together with introduction of new ISBT 128 codes.
Defining new codes and labels for new pools (apheresis pools).</td></tr>
<tr><td>Donor Blood Typing</td><td colspan="3">Changing scheduled time for blood donor typing, due to a new demand/procedure.</td></tr>
</table>
